# Supplementary material for: Cardiac Disease Alters Myocardial Tissue Levels of Epoxyeicosatrienoic Acids and Key Proteins Involved in Their Biosynthesis and Degradation
Source: Int J Mol Sci. 2022 Oct 17;23(20):12433. doi: 10.3390/ijms232012433 (PMC9604309; doi:10.3390/ijms232012433)
Supplement: Supplementary file 1 [file ijms-23-12433-s001.zip › ijms-1864271-supplementary.pdf]

Supplementary Table S1. List of peptides and mass spectrometric parameters for CYP2J2, EPHX2, POR, LPCAT1, PLA2G4A, PLA2G7, HMOX1, BSA, Na^{+}/K^{+}-ATPase, and LDH protein quantification.

Protein	Peptide sequence	Light/Heavy	Parent ion	Product ion	Retention time (min)	CE (eV)	DP (V)
CYP2J2	VIGQGQQPSTAAR	Light	656.85	915.46	8.3	32.5	69
				730.38			
				602.33			
		Heavy	661.86	925.47			
				740.39			
				612.33			81.1
	EVTVDTTLAGYHLPK	Light	548.63	785.43	14.2	22.4	61.1
				714.39			
				608.32			
		Heavy	551.3	793.44			
				722.41			
				612.33			71.5
	LLDEVTYLEASK	Light	690.87	910.49	14.8	33.7	71.5
				811.42			
				710.37			
		Heavy	694.87	918.5			
				819.43			
				718.39			70.9
EPHX2	VC[CAM]EAGGLFVNSPEEPSLSR	Light	683.33	914.46	14.5	30	70.9
				559.32			
				457.73			
		Heavy	686.67	924.47			
				569.33			
				462.74			57.8
	GLLNDAFQK	Light	503.27	835.43	13.9	22	57.8
				722.35			
				608.3			
		Heavy	507.28	843.45			
				730.36			
				616.32			72.2
	ASPSEVVFLDDIGANLKPAR	Light	700.38	764.93	17.4	30.7	72.2
				715.39			
				647.69			
		Heavy	703.71	769.93			
				720.4			
				651.02			55.9
POR	FAVFGLGNK	Light	476.77	734.42	15.5	21	55.9
				635.35			
				488.28			
		Heavy	480.77	742.43			
				643.37			
				496.3			57.8
	YYSIASSSK	Light	503.25	842.43	9.7	25	57.8
				679.36			
				592.33			
		Heavy	507.26	850.44			

				687.38			
				600.34			61.4
LPCAT1	NPALYASNVR	Light	552.79	893.48	10.4	61.4	28.8
				822.45			
				709.36			
		Heavy	557.80	903.49			
				832.46			
				719.37		48	28.8
	TC[CAM]LITFKPGAFIP-GAPVQPVVLR	Light	827.81	1132.68	18.3	81.5	37.6
				907.57			
			827.81	566.85			
		Heavy	831.14	1142.69			
				917.58			
				571.85		58.5	
PLA2G4A	IDPYVFDR	Light	512.76	796.40	13.7	58.5	22.3
				536.28			
				398.70			
		Heavy	517.76	806.41			
				546.29			
				403.71		83.1	
PLA2G7	YPLVVFSHGLGAFR	Light	521.62	651.87	17.3	59.1	26
			521.62	595.33			
			521.62	467.27			
		Heavy	524.96	656.87			
			524.96	600.33			
			524.96	470.60		53.1	
	.ASLAFLQK	Light	439.26	719.45	13.7	53.1	20
			439.26	606.36			
			439.26	535.32			
		Heavy	443.27	727.46			
			443.27	614.38			
			443.27	543.34		75.8	
HMOX1	TEPELLVAHAYTR	Light	750.40	930.52	13	75.8	35.9
			750.40	817.43			
			750.40	635.35			
		Heavy	755.40	940.52			
			755.40	827.44			
			755.40	640.36		79.2	
BSA	AEFVEVTK	Light	461.75	722.41	10.7	21.5	54.8
				575.34			
				476.27			
		Heavy	465.75	730.42			
				583.35			
				484.29			63.6
	LVNELTEFAK	Light	582.32	951.48	14.4	25	63.6
				595.31			
				218.15			
		Heavy	586.33	959.49			
				603.32			
				226.16			51.3
NaKATPase	AAVPDAVGK	Light	414.23	685.39	8.9	23.8	51.3

				586.32			
				489.27			
		Heavy	418.24	693.4			
				594.33			
				497.28			45
	LSLDELHR	Light	328.18	435.23	11.7	15.4	45
				391.71			
		Heavy	331.52	440.23			
				396.72			48.2
LDH	LNLVQR	Light	371.73	515.33	10.3	22.2	48.2
				228.13			
		Heavy	376.74	525.34			
				228.13			56.1
	FIIPQIVK	Light	479.31	697.46	16.4	21.1	56.1
				584.38			
		Heavy	483.32	705.47			
				592.39			

Supplementary Table S2. Liquid chromatography gradient used to separate different peptides from digested protein of interest.

Time (min)	Flow rate (mL/min)	% A	% B
**0**	0.3	97	3
**4.0**	0.3	97	3
**8.0**	0.3	87	13
**18.0**	0.3	70	30
**22.9 .**	0.3	60	40
**23.0**	0.3	40	60
**23.1**	0.3	97	3
**27**	0.3	97	3

Supplementary Table S3. Mass transitions and mass spectrometer parameters for terfenadine metabolites and internal standard, midazolam.

	Mass Transition	DP (V)	CE (V)
Hydroxy terfenadine	488.3 > 452.2	100	40
Carboxy terfenadine	502.4 > 466.3	50	30
Azacyclonol	267.8 > 91.15	91	39
Midazolam	326.0 > 291.2	90	40
